# Nutritional knowledge, sociodemographic, and lifestyle factors as determinants of diet quality – a Polish population-based study

**DOI:** 10.3389/fpubh.2025.1613598

**Published:** 2025-08-21

**Authors:** Alicja Kucharska, Beata Irena Sińska, Mariusz Panczyk, Piotr Samel-Kowalik, Filip Raciborski, Aneta Czerwonogrodzka-Senczyna, Iwona Boniecka, Iwona Traczyk

**Affiliations:** ^1^Department of Human Nutrition, Faculty of Health Sciences, Medical University of Warsaw, Warsaw, Poland; ^2^Department of Education and Research in Health Sciences, Medical University of Warsaw, Warsaw, Poland; ^3^Department of Prevention of Environmental Hazards, Allergology and Immunology, Faculty of Health Sciences, Medical University of Warsaw, Warsaw, Poland; ^4^Department of Clinical Dietetics, Faculty of Health Sciences, Medical University of Warsaw, Warsaw, Poland; ^5^Department of Public Health, Faculty of Health Sciences, Medical University of Warsaw, Warsaw, Poland

**Keywords:** diet quality, nutritional knowledge, sociodemographic disparities, Poland, latent class analysis

## Abstract

**Introduction:**

Nutritional knowledge is a recognized determinant of dietary behaviors, though its impact may vary with sociodemographic and lifestyle factors. To capture such interactions and population variability, advanced methods like moderation and latent class analyses are needed. This study aimed to examine the relationship between nutritional knowledge and diet quality among Polish adults, accounting for socioeconomic determinants, and to identify subgroups at risk of poorer dietary patterns.

**Methods:**

A cross-sectional study was conducted using data from two nationally representative surveys (2017–2020), totaling 4,000 adults. Nutritional knowledge was measured with the validated KomPAN questionnaire, and diet quality was assessed with the Diet Quality Index (DQI). Associations were tested via linear regression, moderation analysis explored interactions between knowledge and demographics, and latent class analysis (LCA) identified dietary lifestyle subgroups.

**Results:**

The mean DQI score was −0.79 (SD = 13.40). Higher nutritional knowledge (*β* = 0.87, *p* < 0.001) and higher education were positively associated with diet quality. Women and older adults had better diets; smoking and alcohol consumption were linked to poorer outcomes. Multivariate models confirmed nutritional knowledge, sex, and age as independent predictors. The beneficial effect of knowledge was weaker in older adults (*β* = −0.49, *p* < 0.001). LCA revealed three profiles; the poorest diets occurred among younger men with low knowledge and unhealthy behaviors, and younger adults with higher socioeconomic status.

**Conclusion:**

Nutritional knowledge supports better diet quality but may not suffice especially in older adults. Tailored public health strategies are needed for vulnerable groups, including younger men with low knowledge and those with higher socioeconomic status but poor diets.

## 1 Introduction

Diet quality is a key factor affecting public health, and its low level is one of the main risk factors for non-communicable diseases (1). Numerous studies indicated that a properly balanced diet, characterized by the predominance of products of plant origin, including fresh fruits and vegetables, whole grains, legumes, nuts and seeds, while limiting the consumption of highly processed food and animal products, especially fatty and processed meat, reduced the risk of non-communicable diseases such as obesity, type 2 diabetes, cardiovascular diseases and some tumors. A diet with a high supply of saturated and trans-fatty acids, salts and simple sugars, promotes the development of metabolic diseases (2–8).

Nutritional knowledge is an important factor determining the quality of the diet. It enables conscious nutritional decisions and, consequently, healthier eating habits. Research showed that people with greater nutritional knowledge tended to have a higher-quality diet (9–11), consumed better-balanced meals, had a lower BMI, and were less likely to be obese (12). While nutritional knowledge is crucial for improving diet quality, it is not the only factor influencing it, and its impact may be affected by socioeconomic factors and lifestyle (13–16). Age influences diet quality by changing eating habits and evolving nutritional needs. However, evidence in this regard remains inconclusive (17–20). Sex-related differences also appear significant, with women generally demonstrating better diet quality, likely due to physiological, psychological and sociocultural factors. They are more commonly involved in meal preparation, more likely to follow dietary recommendations and seek information on healthy eating (21, 22).

Other important determinants include the place of residence (rural inhabitants often have poorer access to diverse food options); education (which correlates with greater nutritional awareness and healthier choices); and financial status (which may constrain the ability to follow dietary recommendations, particularly in low-income populations). Notably, even among those with adequate nutritional knowledge, unfavorable socioeconomic or environmental conditions may hinder the adoption of healthy eating practices (23).

Furthermore, diet quality may be affected by other lifestyle factors, such as smoking and alcohol consumption. Studies indicated that smokers were more likely to have poorer eating habits, eat fruit and vegetables less often, and have a poorer nutrient intake (24, 25).

Similarly, alcohol consumption might exert a negative impact on diet quality, often leading to poorer food choices and increased health risks (26, 27).

Although the association between nutritional knowledge and diet quality has been extensively explored, significant gaps remain concerning how this relationship is modified by socioeconomic determinants and lifestyle factors. Existing evidence is inconsistent regarding the extent to which sociodemographic variables, such as age, educational attainment, and behavioral practices, may interact with nutritional knowledge to influence dietary patterns. Addressing these uncertainties is crucial for understanding mechanisms underpinning dietary inequalities and for the effective targeting of nutritional interventions aimed at improving public health outcomes, particularly within diverse and potentially vulnerable population subgroups.

This study aimed to evaluate the association between nutritional knowledge and diet quality in the adult population of Poland, explicitly considering socioeconomic and demographic contexts. The specific objectives were to investigate generational differences in diet quality, identify demographic and behavioral subgroups at higher risk of poor dietary patterns, and examine how socioeconomic and lifestyle factors moderate the relationship between nutritional knowledge and diet quality. By elucidating these mechanisms, the study seeks to inform targeted, evidence-based strategies that can effectively reduce dietary inequalities and improve overall nutritional status within the population.

## 2 Materials and methods

### 2.1 Design

The present authors analyzed data derived from two representative cross-sectional surveys examining dietary habits and nutritional status in Polish adults. The surveys were conducted between 2017 and 2020 within the framework of the National Health Program and funded by the Polish Ministry of Health. The combined sample comprised 4,000 participants. A detailed description of the survey methodology was published elsewhere (28).

### 2.2 Sampling

The respondents were selected according to guidelines provided by the European Food Safety Authority (EFSA) (29) using random sampling from a household address database. To improve the representativeness and efficiency, a stratified and clustered sampling approach was employed. Initially, 500 statistical areas (clusters) were randomly selected from 34,633 units with the probability being proportional to the population size. Residential buildings within each cluster were identified using the TERYT-NOBC register and supplementary sources. Subsequently, eight buildings per cluster (four per subgroup) were selected, with reserves being allocated in case of non-participation. One respondent was randomly chosen from each selected building.

### 2.3 Sample size and representativeness

A minimum sample size of approximately 1,067 respondents would provide a margin of error of no more than 3% assuming a proportion of 0.5 and a 95% confidence level. However, the present study used a substantially larger sample of 4,000 individuals, further reducing the margin of error and enabling detailed subgroup analyses (e.g., by age, sex, education). Post-stratification weights were applied to reflect the distribution of sex, age, residence, and education level in the Polish population. They were calibrated using data from the latest National Population and Housing Census (57) conducted by the Statistics Poland (30). The detailed characteristics of the study population were published previously (31).

### 2.4 Data collection

The data were primarily collected through computer-assisted personal interviewing (CAPI), accounting for 90% of all completed interviews. Due to the COVID-19 pandemic, the remaining 10% were conducted via computer-assisted telephone interviewing (CATI). The initial interview lasted approximately 90 min, while the follow-up interview took about 45 min.

### 2.5 Instruments

Nutritional knowledge and diet quality were assessed using the validated Dietary Habits and Nutrition Beliefs Questionnaire (KomPAN), developed by experts from the Committee of Human Nutrition Science of the Polish Academy of Sciences. This tool is widely applied in dietary research conducted in Poland (32).

Nutritional knowledge was evaluated based on participants’ responses to 19 statements concerning food and nutrition, with response options being ‘True’, ‘False’, or ‘Difficult to say’. Correct answers obtained 1 point each, whereas incorrect or ‘Difficult to say’ responses scored 0 points. The total score was used to classify the participants into one of three categories of nutritional knowledge: insufficient (0–6 points), sufficient (7–12 points), or good (13–19 points).

Diet quality was assessed with the use of a simplified version of the Food Frequency Questionnaire (FFQ), comprising 24 questions. The respondents reported the frequency of consuming specific food groups by choosing one of six response categories: never, 1–3 times/month, once/week, several times/week, once/day, or several times/day. To enable quantitative analysis, each response category was converted into daily frequency values (times/day), following the KomPAN methodology (23). Diet quality was evaluated based on the Diet Quality Index (DQI), calculated using two component indices: the Pro-Healthy Diet Index (pHDI-10) and the Non-Healthy Diet Index (nHDI-14). The pHDI-10 included ten food groups considered beneficial for health (wholemeal bread; wholegrain cereals; milk; fermented dairy products; cottage cheese; white meat dishes; fish; legumes; fruits; vegetables). The nHDI-14 comprised fourteen food groups whose high consumption might exert an adverse effect on health (white bread; refined cereals; fast food; fried meat or flour-based dishes; butter; lard; hard cheeses; processed meats or sausages; red meat dishes; sweets; canned meat products; sweetened carbonated or non-carbonated beverages; energy drinks; alcoholic beverages).

The overall Diet Quality Index (DQI) incorporated both health-promoting and potentially harmful dietary components. The pHDI and nHDI indices were calculated by summing the consumption frequencies of relevant food groups, which were then standardized to a 0–100-point scale for comparability.

The Pro-Healthy Diet Index was calculated using the formula:


$$\text{pHDI}=\frac{100}{20}\times \mathrm{sum}\;\text{of daily frequencies of}\;10\;\text{healthy food groups}$$

The Non-Healthy Diet Index was calculated using the formula:


$$\text{nHDI}=\frac{100}{28}\times \mathrm{sum}\;\text{of daily frequencies of}\;14\;\text{unhealthy food groups}$$

The combined DQI was computed as the sum of pHDI (positive values) and nHDI (negative values), balancing the contributions of each component:


$$\begin{array}{c} \mathrm{DQI}=(\frac{100}{20}\times \mathrm{sum}\;\text{of daily frequencies of}\;10\;\text{healthy food groups})\\ -(\frac{100}{28}\times \mathrm{sum}\;\text{of daily frequencies of}\;14\;\text{unhealthy food groups}) \end{array}$$

DQI scores ranged from −100 to 100 points, with higher scores indicating better diet quality characterized by the predominance of beneficial food groups, whereas lower scores reflected poorer diet quality due to the greater consumption of non-recommended foods.

Additionally, the data were collected regarding selected lifestyle factors (smoking, alcohol consumption) and sociodemographic characteristics (sex, age, education level, place of residence, and socioeconomic status).

### 2.6 Statistical analysis

All statistical analyses were conducted using weighted procedures to ensure the representativeness of the broader population structure. Weighting adjustments accounted for potential demographic imbalances and enhanced the generalizability of the findings.

Descriptive statistics were used to summarize the characteristics of the study population. Categorical variables were reported as frequencies and percentages. Differences between age groups (19–64 years vs. ≥65 years) were evaluated using the Chi-squared (*χ*^2^) tests for independence.

To examine the determinants of diet quality measured by the Diet Quality Index (DQI), a series of linear regression analyses was conducted. Ordinary least squares (OLS) estimation was used in both univariate and multivariate models. In the univariate analyses, each predictor was examined separately in relation to the DQI score. The predictors included sex, age group, nutritional knowledge, education level, place of residence, financial status, smoking status, and alcohol consumption. The multivariate model included all predictors simultaneously to estimate their independent associations with the DQI, while adjusting for potential confounding.

Standardized regression coefficients (*β*) and 95% confidence intervals (CIs) were reported. Prior to interpreting the results, standard linear regression assumptions had been assessed, including linearity, homoscedasticity, the normality of residuals, and the absence of multicollinearity. Diagnostic procedures included the visual inspection of residual plots and Q-Q plots, the analysis of Cook’s distance, leverage, and standardized residuals to identify potential outliers and influential data points.

Moderation analyses were performed to assess a potential effect modification by age groups (<65 vs. ≥65 years). Interaction terms between the age group and selected predictors (nutritional knowledge, education, and alcohol consumption) were included in the regression models. The statistical significance of interaction effects was evaluated based on the interaction coefficients and their 95% CIs.

Latent class analysis (LCA) was employed to identify distinct subgroups within the population based on patterns of diet quality, sociodemographic attributes, and health-related behaviors. A series of models with increasing numbers of latent classes was estimated. Model fit was evaluated using the Akaike Information Criterion (AIC), Bayesian Information Criterion (BIC), and the Sample-Size Adjusted BIC (SABIC). In addition, entropy values were assessed to evaluate classification accuracy. The optimal number of classes was determined by combining statistical criteria with theoretical interpretability and parsimony.

All statistical analyses were performed using STATISTICA™ version 13.3 (TIBCO® Software Inc., Palo Alto, CA, USA). A two-tailed significance level of *α* = 0.05 was applied in all hypothesis testing.

## 3 Results

### 3.1 Study group characteristics

The study included 4,000 adult participants, with the equal size (*n* = 2000) of the age groups of 19–64 years and ≥65 years. Women predominated in the younger age group (58.9%), whereas sex-related proportions were balanced in seniors (*p* < 0.001). Most participants lived in urban areas, with no significant differences occurring between age groups (*p* = 0.115). Seniors were characterized by higher education levels (*p* < 0.001) and were more likely to rate their financial situation as good (30.1% vs. 9.9%, *p* < 0.001). Current smoking was more prevalent in seniors compared to younger adults (32.5% vs. 19.3%, *p* < 0.001), while past smoking was more frequent in the younger age group (27.3% vs. 16.8%, *p* < 0.001). Regular alcohol consumption was higher in seniors (45.0% vs. 20.1%, *p* < 0.001). Detailed characteristics of the study group are presented in Table 1.

**Table 1:** Study group characteristics.

Variable	Total	Adults 19–64 years		Seniors 65+ years		*χ* ^2^	*p*-value*
	*N*	*N*	%	*N*	%		
Sex							
Female	2,178	1,177	58.9	1,001	50.0	31.2	<0.001
Male	1822	823	41.1	999	50.0		
Place of residence							
Village	1,378	645	35.4	733	39.1		
Town (up to 20 thousand inhabitants)	505	254	13.9	251	13.4	5.9	0.115
Town (20–100 thousand)	737	367	20.1	370	19.7		
City (over 100 thousand)	1,078	556	30.5	522	27.8		
Education							
Primary/junior high school/vocational	1884	1,252	62.6	632	31.6	403.0	<0.001
Secondary (general or technical)	1,627	615	30.8	1,012	50.6		
Tertiary (bachelor’s degree, engineering studies, master’s degree)	489	133	6.7	356	17.8		
Financial situation**							
Poor	395	288	14.4	107	5.3	306.0	<0.001
Average	2,804	1,514	75.7	1,290	64.5		
Good	801	198	9.9	603	30.1		
Current smoking							
No	2,964	1,614	80.7	1,350	67.5	90.8	<0.001
Yes	1,036	386	19.3	650	32.5		
Smoking in the past							
No	2,296	1,173	72.7	1,123	83.2	46.5	<0.001
Yes	668	441	27.3	227	16.8		
Current alcohol drinking							
Less often than once a week	2,700	1,599	80.0	1,101	55.0	283.0	<0.001
At least once a week	1,300	401	20.1	899	45.0		

*Chi-squared test. **Financial situation was self-assessed by the respondents and categorized as follows: “good” – having sufficient resources for all expenses without the need to save, including major purchases, or the ability to save some money; “average” – having enough money for daily expenses but not for additional spending; “poor” – having to forgo many needs to afford basic living expenses, or lacking resources even for the most urgent necessities.

### 3.2 Nutritional knowledge and diet quality

**Figure 1:**
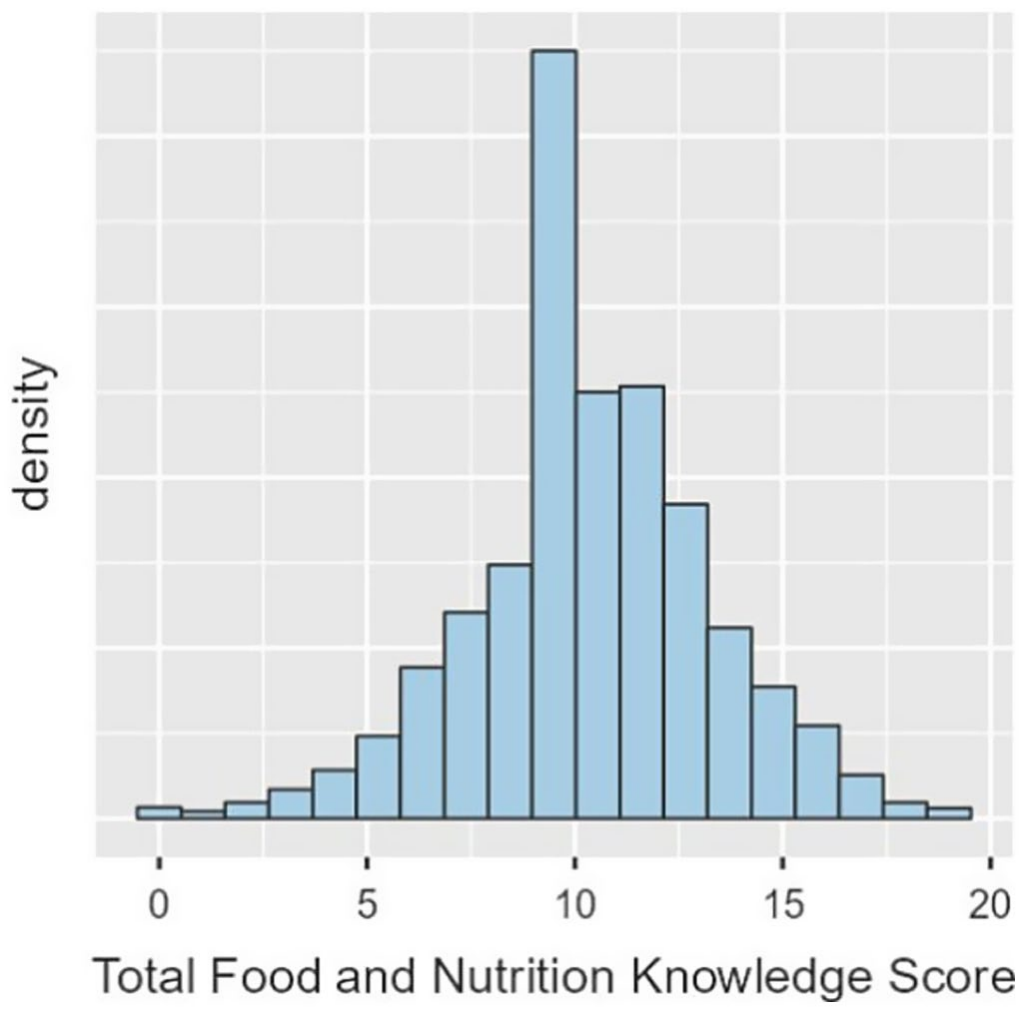
Distribution of nutritional knowledge scores in the total study population.

The mean nutritional knowledge score in the total study population was 10.4 points (SD = 3.13), with the median of 11 points (Figure 1). Detailed data on responses to individual nutrition knowledge items are provided in the Supplementary material 1.

**Figure 2:**
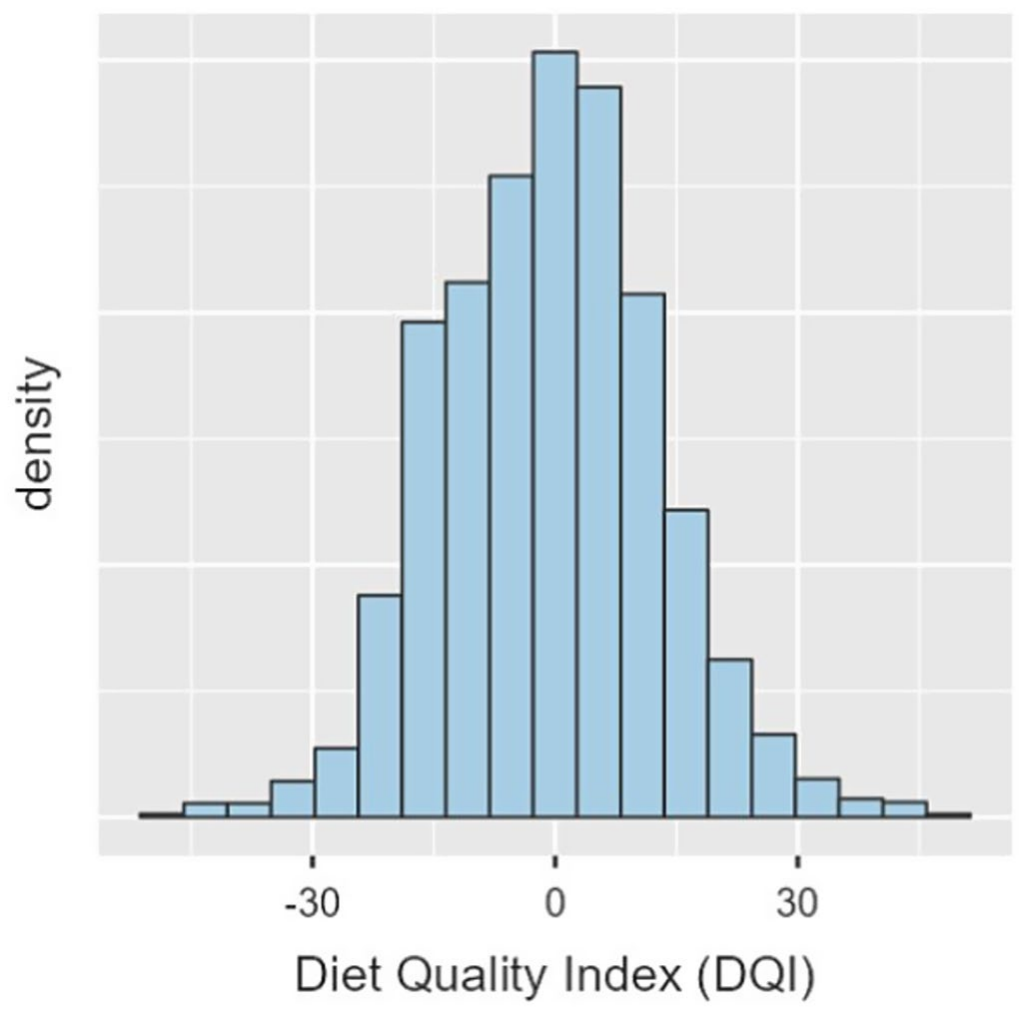
Distribution of Diet Quality Index (DQI) scores in the total study population.

The mean Diet Quality Index (DQI) score was −0.79 points (SD = 13.40), with the median of −0.5 points (Figure 2).

### 3.3 Determinants of diet quality

The predictors of the analyzed factors on diet quality were assessed using univariate and multivariate linear regression analyses, as presented in Table 2.

**Table 2:** Summary of regression analyses predicting the Diet Quality Index (DQI).

Predictor	Univariate analysis		Multivariate analysis		Moderation analysis*		Interaction term**	
	*β* (95% CI)	*p*-value	*β* (95% CI)	*p*-value	*β* (95% CI)	*p*-value	*β* (95% CI)	*p*-value
Senior vs. Non-senior	0.21 (0.15; 0.27)	<0.001	0.18 (0.12; 0.24)	<0.001	0.18 (0.12; 0.24)	<0.001	–	–
Male vs. Female	−0.79 (−0.85; −0.74)	< 0.001	−0.58 (−0.63; −0.52)	<0.001	−0.92 (−1.00; −0.84)	<0.001	0.29 (0.18; 0.41)	<0.001
Dietary knowledge (Sufficient vs. Insufficient)	0.36 (0.23; 0.48)	<0.001	0.26 (0.14; 0.37)	<0.001	0.52 (0.33; 0.70)	<0.001	−0.27 (−0.52; −0.02)	0.036
Dietary knowledge (Good vs. Insufficient)	0.87 (0.74; 1.01)	<0.001	0.61 (0.49; 0.72)	<0.001	1.14 (0.95; 1.33)	<0.001	−0.49 (−0.75; −0.23)	<0.001
Education (Secondary vs. Primary)	0.28 (0.22; 0.35)	<0.001	0.23 (0.17; 0.29)	<0.001	0.47 (0.37; 0.56)	<0.001	−0.13 (−0.27; 0.00)	0.052
Education (Tertiary vs. Primary)	0.63 (0.53; 0.72)	<0.001	0.51 (0.42; 0.60)	<0.001	0.89 (0.77; 1.02)	<0.001	−0.32 (−0.53; −0.11)	0.003
Urban vs. Rural residence	0.13 (0.07; 0.20)	<0.001	0.03 (−0.02; 0.09)	0.221	0.14 (0.06; 0.23)	0.001	−0.05 (−0.17; 0.08)	0.479
Financial status (Average vs. Poor)	0.07 (−0.04; 0.17)	0.200	0.03 (−0.06; 0.13)	0.465	0.13 (−0.07; 0.32)	0.203	−0.02 (−0.25; 0.21)	0.870
Financial status (Good vs. Poor)	0.03 (−0.09; 0.15)	0.607	0.11 (0.00; 0.22)	0.046	0.17 (−0.04; 0.37)	0.105	−0.06 (−0.34; 0.21)	0.648
Currently smoking (Yes vs. No)	−0.396 (−0.47; −0.33)	<0.001	−0.14 (−0.20; −0.07)	<0.001	−0.38 (−0.47; −0.29)	<0.001	0.03 (−0.11; 0.18)	0.651
Alcohol consumption (Yes vs. No)	−0.600 (−0.66; −0.54)	<0.001	−0.32 (−0.38; −0.26)	<0.001	−0.63 (−0.72; −0.55)	<0.001	0.14 (0.00; 0.27)	0.047

*Moderation analysis examines whether the relationship between a predictor and the Diet Quality Index (DQI) depends on a third variable (moderator: Senior vs. Non-senior). **A statistically significant interaction term indicates that the effect of a predictor on the DQI differs depending on the level of the moderator. β, standardized regression coefficient; 95% CI, 95% confidence interval.

In the univariate analysis, sex, nutritional knowledge, education level, and lifestyle factors emerged as significant predictors of diet quality. Women had higher DQI scores than men (*β* = −0.79; 95% CI: −0.85 to −0.74; *p* < 0.001), and individuals with good nutritional knowledge demonstrated significantly better diet quality than those with insufficient knowledge (*β* = 0.87; 95% CI: 0.74–1.01; *p* < 0.001). Similarly, higher educational attainment was positively associated with diet quality (*β* = 0.63; 95% CI: 0.53–0.72; *p* < 0.001).

**Figure 3:**
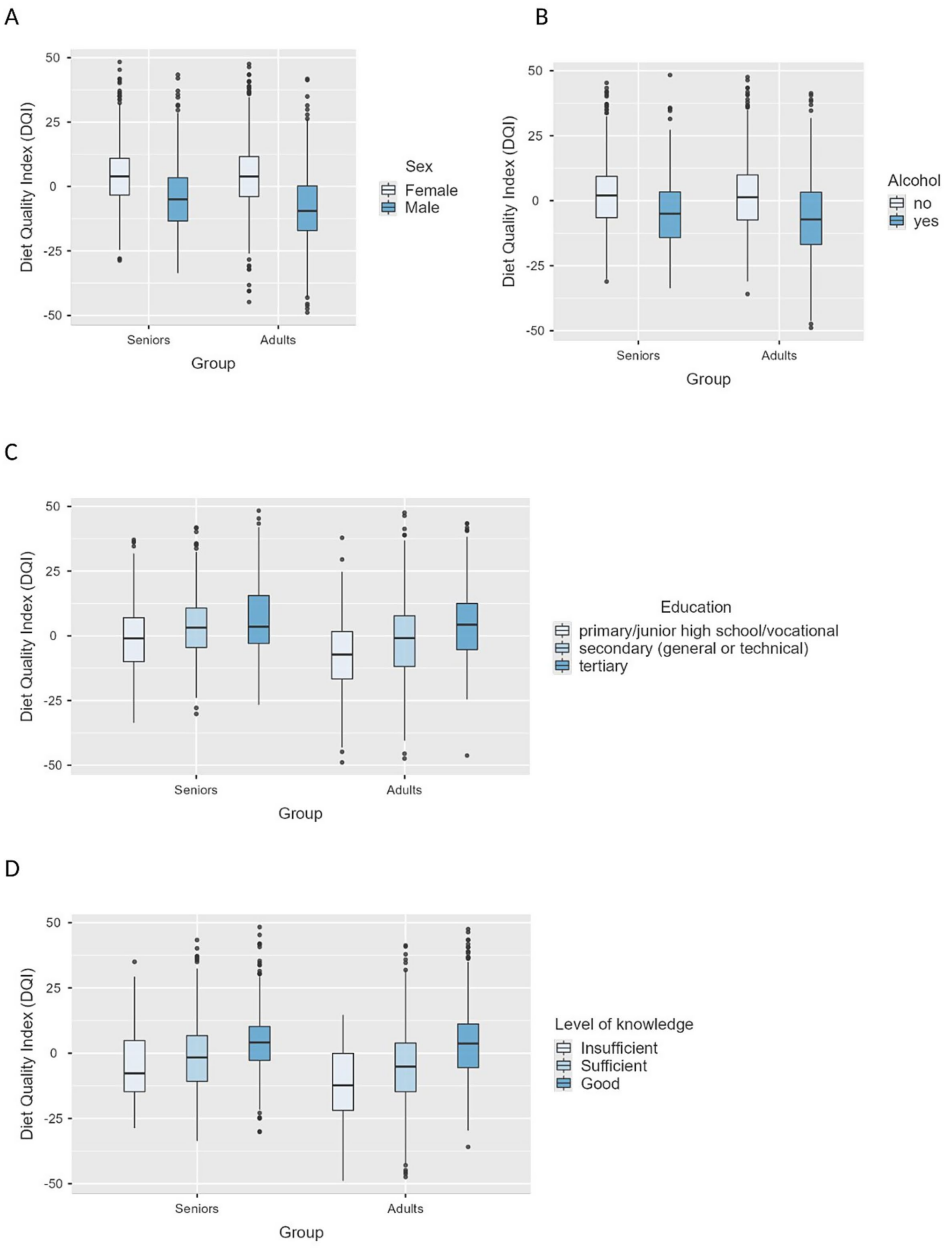
Boxplots of Diet Quality Index (DQI) scores by sex **(A)**, alcohol consumption **(B)**, education level **(C)**, and nutritional knowledge level **(D)**.

Significantly lower DQI scores were observed among smokers (*β* = −0.40; 95% CI: −0.47 to −0.33; *p* < 0.001) and individuals who reported alcohol consumption (*β* = −0.60; 95% CI: −0.66 to −0.54; *p* < 0.001). Older adults (≥65 years) were characterized by a higher diet quality compared to younger adults (*β* = 0.21; 95% CI: 0.15–0.27; *p* < 0.001). Corresponding results are illustrated in Figure 3.

After adjusting for all predictors in the multivariate regression model, some associations observed in the univariate analysis were attenuated. Sex remained a significant predictor of diet quality, with men exhibiting lower DQI scores than women (*β* = −0.58; 95% CI: −0.63 to −0.52; *p* < 0.001). Nutritional knowledge continued to show a positive association with diet quality, although the effect size was reduced in comparison with the univariate model (*β* = 0.61; 95% CI: 0.49–0.72; *p* < 0.001). Older adults maintained significantly better diet quality than younger participants (*β* = 0.18; 95% CI: 0.12–0.24; *p* < 0.001).

In the multivariate model, the place of residence was not significantly associated with diet quality (*p* = 0.221). Financial situation had a limited effect; only the comparison between good and poor financial status showed a weak but statistically significant association (*β* = 0.11; *p* = 0.046).

**Figure 4:**
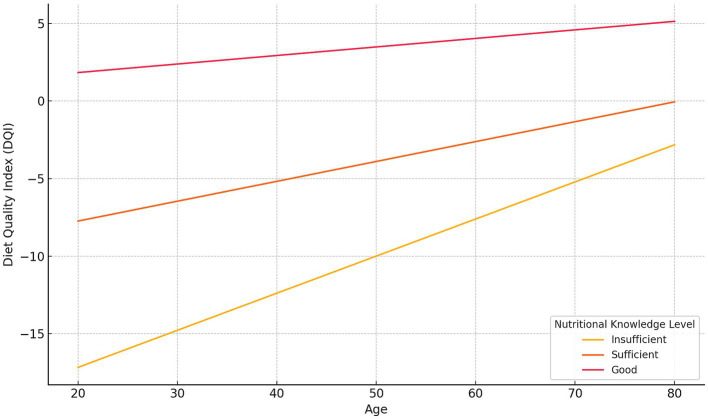
The moderating effect of knowledge level on the relationship between age and the Diet Quality Index (DQI). Regression lines show predicted DQI values for three knowledge levels—Insufficient, Sufficient, and Good—based on the estimated intercept and slope coefficients from the moderation model.

**Figure 5:**
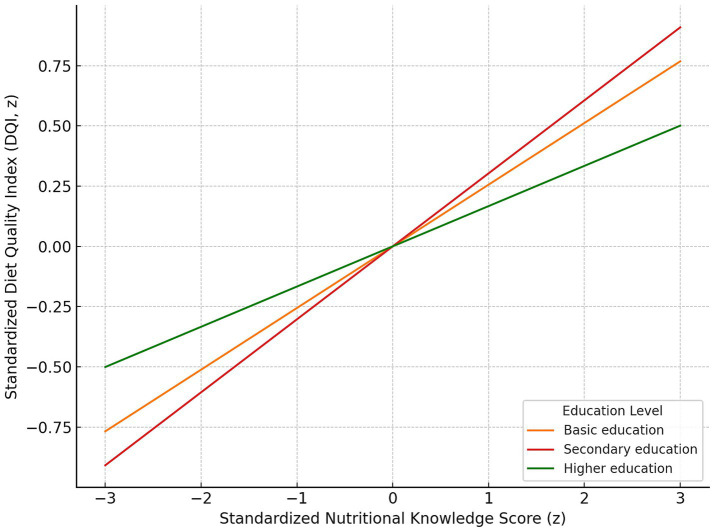
Moderating effect of education level on the relationship between standardized nutritional knowledge score and standardized Diet Quality Index (DQI). Regression lines depict predicted DQI across z-scored knowledge values for Basic education (orange), Secondary education (red) and Higher education (green). The slope is steepest for the Secondary education group and flattest for the Higher education group, indicating the strongest and weakest associations between knowledge and DQI, respectively.

Moderation analysis revealed significant interactions between age and both nutritional knowledge and the education level in relation to diet quality. The positive effect of good nutritional knowledge on diet quality was weaker in older adults (*β* = −0.49; 95% CI: −0.75 to −0.23; *p* < 0.001) (Figure 4). A similar moderation pattern was observed for higher education (*β* = −0.32; 95% CI: −0.53 to −0.11; *p* = 0.003) (Figure 5).

Lifestyle factors remained significantly associated with diet quality. Lower DQI scores were found in individuals who smoked (*β* = −0.38; *p* < 0.001) and those who consumed alcohol (*β* = −0.63; *p* < 0.001). A significant interaction between age and alcohol consumption was also observed (*β* = 0.14; *p* = 0.047).

### 3.4 Latent class profiling

Using Latent Class Analysis (LCA), three distinct population profiles were identified, differing in diet quality, sociodemographic characteristics, and health-related behaviors. Detailed information on the class extraction procedure is provided in Supplementary material 2.

The first and largest class (Profile 1, *N* = 1,619) was characterized by a higher proportion of women aged 65 years and older, residing predominantly in urban areas. Individuals in this group had high levels of nutritional knowledge, reported the best diet quality, and demonstrated health-promoting behaviors, including lower rates of smoking and alcohol consumption. They also reported higher educational attainment and their self-reported good financial status was described as good.

Profile 2 (*N* = 1,135) represented a group of older adults characterized by lower educational levels, living mostly in rural areas. Despite poorer financial status, this group displayed moderate diet quality and a relatively low prevalence of risky behaviors, such as smoking and alcohol consumption.

The third class (Profile 3, *N* = 1,246) consisted primarily of younger individuals, with the predominance of men. This group was marked by the lowest levels of nutritional knowledge and diet quality, alongside the highest rates of smoking and alcohol use. Interestingly, despite reporting relatively favorable financial conditions, their dietary patterns were the least healthful among all identified classes.

**Figure 6:**
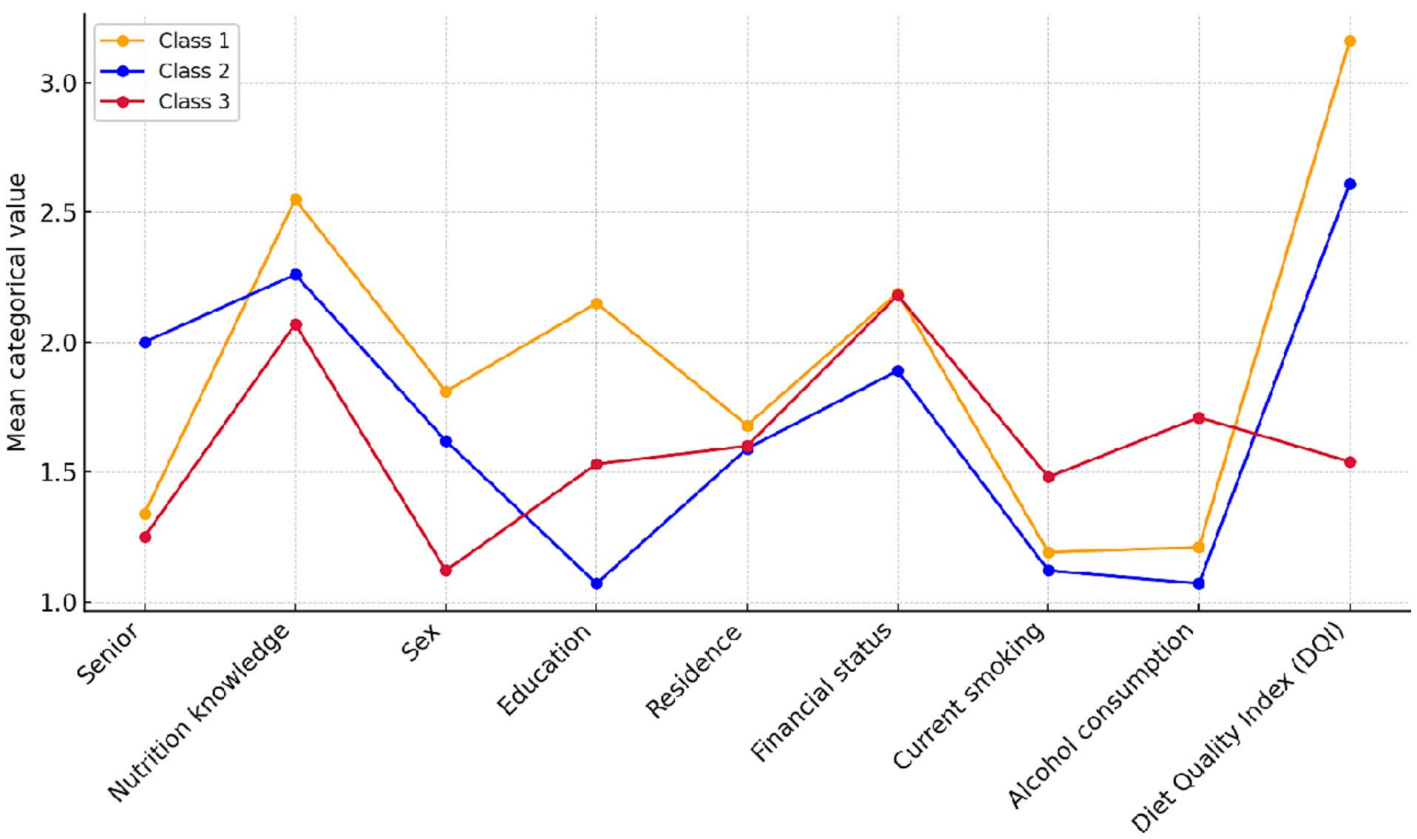
Profiles of the three latent classes based on mean categorical values of nine variables: age group (senior status), nutritional knowledge, sex, education level, place of residence, financial status, current smoking, alcohol consumption, and the Diet Quality Index (DQI). The values reflect mean category scores assigned to each characteristic.

Figure 6 illustrates the profile characteristics of the three latent classes based on nine variables: age group, nutritional knowledge, sex, education level, place of residence, financial situation, current smoking status, alcohol consumption, and the overall Diet Quality Index (DQI).

## 4 Discussion

Diet quality is widely recognized as a crucial determinant of population health, influenced by a complex interplay of individual, social, and behavioral factors. Among these determinants, nutritional knowledge plays a pivotal role by enabling individuals to make informed and health-promoting dietary choices. However, the translation of nutritional knowledge into practice is influenced by a variety of sociodemographic and lifestyle factors, including age, sex, educational attainment, economic status, and health-related behaviors. The interactions among these variables form a nuanced network of influences, in which specific factors may mutually reinforce or diminish the positive effects of nutritional knowledge on dietary outcomes. Understanding these intricate relationships is essential for the development of effective, tailored public health strategies that aim to enhance diet quality across diverse population groups.

Nutritional knowledge includes both knowledge of the principles and terminology of nutrition, as well as attitudes toward nutrition that shape the motivations, beliefs and emotions associated with food choices. Both these aspects may influence the actual eating behaviors and diet quality. However, their significance may vary depending on social and demographic factors (33).

The present study was conducted to analyze the relationship between nutritional knowledge and diet quality comprising the broad sociodemographic context in order to better understand the mechanisms which shape eating behaviors in the population of Polish adults.

During the first stage of the analysis, univariate models were used to assess the basic relationships between the quality of diet and selected variables, such as nutritional knowledge, sex, age, level of education, financial situation and lifestyle factors. The present results confirmed that individuals with greater nutritional knowledge were characterized by better diet quality, which was expressed as higher DQI scores. A similar relationship was found in relation to the level of education. Sex also played an important role, i.e., the diets of women were characterized by a higher quality compared to those of men. People who smoked tobacco and consumed alcohol on a regular basis were characterized by lower diet quality compared to non-smokers and non-drinkers. The difference between the age groups regarding diet quality (seniors reported a better quality of their diets than the younger group of adults) was also an interesting finding. The obtained results are consistent with previous studies that emphasized the importance of the abovementioned factors as key determinants of diet quality (11–29, 34–37).

In order to determine the independent strength of individual predictors and to examine their mutual interactions, multivariate models, moderation analysis and latent class analysis were subsequently used. The results of those analyses showed that nutritional knowledge remained an important predictor of diet quality, but its impact was modified by age, sex and lifestyle factors, such as alcohol consumption or smoking. The weakening of the impact of nutritional knowledge in the older adult was an interesting outcome. It strongly correlated with the quality of diet in younger adults, while in seniors the impact was weaker. Latent class analysis revealed that the quality of the diet of seniors was not directly correlated with the level of nutritional knowledge, which suggests that other factors determining dietary choices played a key role in this age group. According to the literature, seniors are characterized by a greater stability of eating patterns, which means that their dietary choices are more strongly conditioned by long-term eating habits than current nutrition education. Furthermore, their nutritional decisions may result from their health status and the sociocultural context. Research by Mahony et al. (38) and Latorre et al. (39) revealed that older people were more likely to follow established eating habits, based on traditional dishes and proven dietary patterns. This phenomenon results not only from preferences, but also from physiological factors, i.e., changes in the perception of taste and smell, dental problems and health restrictions may affect food choices, making seniors stick to well-known products and ways of preparing meals. It was shown that difficulties in eating and gastrointestinal complaints constituted risk factors for poor diet quality (40, 41). Numerous older people may consider adherence to established dietary patterns as a form of autonomy and control over their own health (42). This behavioral rigidity, combined with health-related constraints, may explain why nutrition education is less important than in younger age groups. At the same time, attachment to known dietary habits may in some cases hinder adaptation to new dietary recommendations, which may pose a significant challenge to the effectiveness of educational interventions addressed to this population group. The psychosocial context is another important factor influencing dietary behaviors of older adults. Older individuals who maintain close family relationships or engage in active communities are less likely to skip meals, eat more regularly, and have better access to diverse foods. Social support, both emotional and practical, promotes healthier eating patterns, as confirmed by studies showing the positive impact of participation in recreational activities, group meetings, and community-based engagement on diet quality in older adults (43). Chronic illnesses, which are common in older populations, may be a significant motivator for adhering to dietary recommendations. Even with limited nutritional knowledge, such actions are often undertaken to maintain health or slow disease progression. According to Muellers et al., individuals with chronic conditions such as chronic obstructive pulmonary disease (COPD) (44) tend to rely primarily on trust in medical authority, which may explain the weaker effect of nutritional knowledge on diet quality in this group. Despite the importance of these determinants, numerous barriers still hinder the maintenance of a high-quality diet in older age. This applies particularly to individuals with a lower socioeconomic status, limited social support, and restricted access to healthy foods, i.e., those who are more likely to consume less nutritious diets (45).

Although sex remained an important factor differentiating the quality of the diet, multifactorial analysis showed that its impact weakened after comprising other variables, such as the level of education and lifestyle. This may suggest that part of the sex-related effect is indirectly related to other factors – women are more likely to have higher education and are less likely to engage in risky health behaviors such as smoking or excessive alcohol consumption (46, 47). Given these variables, sex determined diet quality to a lesser extent, which indicates that differences between women and men in this respect might result not only from biological conditions, but also from the socioeconomic context and differences in health behaviors. Additional conclusions are drawn from latent class analysis, which showed that women were more likely to belong to groups with a better quality of diet, especially if they had a higher level of education and a stable financial situation. The profile characterized by health-promoting behaviors (class 1) was characterized by a high level of nutritional knowledge and the best quality of the diet. It was dominated by women. The least favorable profile (class 3), associated with poor diet quality and risky health behaviors such as smoking and excessive alcohol consumption, was dominated by men. Research on sex-related differences in food choices showed that women were more likely to select healthier foods and limit their consumption of highly processed foods (48, 49). Women were more likely to undertake actions related to health care, which may result from the traditionally assigned role of health care providers in the family (47). Education seems to be the key factor reducing such differences. The impact of sex on the quality of diet was much weaker in people with a higher level of education (46).

Lifestyle, including alcohol consumption and smoking, remained one of the strongest predictors of diet quality. However, multifactorial analysis demonstrated that its impact varied depending on age and socioeconomic factors. The negative correlation between smoking and diet quality was stable across the groups. Smokers achieved significantly lower DQI scores regardless of age, which suggests that this habit was strongly associated with a less healthy lifestyle. The results of previous research indicated that smokers were less likely to follow dietary recommendations, consumed lower amounts of vegetables, fruits and products rich in fiber, which may explain the persisting relationship between smoking and poor diet quality (50). However, a significant interaction with age was observed in case of alcohol consumption. The negative impact of alcohol on diet quality was stronger in younger adults, while in the older population, this effect was weakened. This may suggest differences in consumption. Episodes of heavy drinking were more common in the younger group, while in seniors, alcohol was found to be consumed in a more “cultural” and moderate way, often accompanied by meals, which might minimize its negative consequences for the quality of the diet (51). It was also confirmed with the latent class analysis, in which individuals with the highest quality diets were less likely to undertake risky health behaviors, while high alcohol consumption and smoking were more common in those with the lowest DQI scores. Population profiling in terms of lifestyle showed that seniors with a high level of education and a stable financial situation were more likely to be characterized by low alcohol and tobacco consumption and the highest diet quality. As regards older people with a lower level of education, the quality of diet was moderate, but risky health behaviors were observed less commonly. The least favorable profile was dominated by young adults, mostly men, who had the lowest quality of diet. At the same time, they consumed alcohol and smoked cigarettes more often, despite their relatively good financial situation. The results indicate that in the older population, the quality of diet may remain at a relatively high level even with a lower level of nutritional knowledge, as long as the lifestyle remains healthy. Conversely, regardless of other factors, negative habits such as smoking and excessive alcohol consumption may have a stronger impact on diet quality in the younger group of adults.

Multivariate analysis showed that financial status and the place of residence had a limited impact on the quality of diet. Although the initial analyses suggested the significance of such factors, their importance weakened after comprising other variables, which indicates that they may mainly have an indirect effect through the level of education and lifestyle. Financial status is not a sufficient predictor of diet quality, as both high-income and lower-income individuals may follow both healthy and less healthy eating patterns (52). Higher income may increase the availability of healthy foods. At the same time, it may promote a greater consumption of highly processed products. Individuals with lower financial status may be more likely to follow traditional dietary patterns, which, despite limited access to a variety of products, are not always unfavorable in terms of nutritional quality (53).

Similarly, the place of residence had no significant impact on the quality of the diet after comprising other factors. Initial analyses suggested some differences between rural and urban inhabitants. However, it turned out that the differences may result from education and financial status. Although urban residents have an easier access to diverse food, they are more likely to consume highly processed foods. In contrast, rural residents, despite restrictions regarding the availability of some products, more commonly rely on homemade and less processed meals (54).

Further insights emerged from the latent class analysis conducted in this study. Participants with lower socioeconomic status were predominantly associated with groups characterized by moderate diet quality but demonstrated a lower prevalence of risky health behaviors, such as excessive alcohol consumption. Conversely, younger participants with a higher socioeconomic status were more frequently classified into groups exhibiting poorer dietary patterns, suggesting that increased financial resources did not necessarily lead to healthier food choices. These findings are consistent with previous research highlighting the complex relationship between socioeconomic status and dietary habits. Other studies similarly reported varied relationships, where lower income groups might consume more processed foods in certain contexts, whereas in other regions, traditional diets consumed by economically disadvantaged populations might actually promote healthier dietary behaviors (14, 55). This complexity underscores the necessity for nuanced, context-specific approaches to nutritional interventions and public health policies aimed at improving diet quality across diverse socioeconomic groups.

### 4.1 Public health implications

The findings of this study suggest that effective public health policies aimed at improving diet quality should extend beyond nutritional education alone, explicitly incorporating broader socioeconomic contexts and addressing generational differences. Nutritional knowledge, although essential, does not consistently translate into healthier dietary behaviors, particularly among older adults whose dietary habits are often deeply ingrained. Therefore, interventions targeting older populations should comprise approaches that account for established routines and cultural dietary preferences.

In contrast, younger populations display more dynamic and changeable dietary patterns, often influenced by modifiable behavioral risk factors such as smoking and excessive alcohol consumption. For these groups, interventions should integrate nutritional education with broader strategies aimed at reducing health-risk behaviors, thereby fostering holistic improvements in lifestyle and dietary choices.

The latent class analysis further highlighted those younger men with lower educational attainment and poorer financial conditions exhibited the lowest diet quality, emphasizing the necessity for tailored interventions. Strategies should move beyond merely addressing economic barriers to actively cultivating nutritional competencies, supporting informed dietary decision-making, and promoting sustainable healthy eating habits. Additionally, educational interventions may play a significant role in reducing observed gender disparities in diet quality, underscoring the need for health programs specifically designed to encourage and support men, particularly those with lower educational levels, in adopting healthier dietary practices. These comprehensive, context-sensitive approaches can effectively reduce dietary inequalities, enhance overall population nutrition, and ultimately improve public health outcomes.

### 4.2 Strengths and limitations

One of the main strengths of the study is the use of a large, representative sample of Polish adults, which allows the generalization of the results to the population and increases their credibility. Moreover, validated research tools such as the KomPAN questionnaire and the DQI index were used, enabling a reliable assessment of nutritional knowledge and diet quality. The reproducibility of the KomPAN tool was confirmed in a large Polish sample (*n* = 954; aged 15–65), demonstrating moderate to very good test–retest agreement across lifestyle factors (*κ* = 0.42–0.96) and nutrition knowledge items (*κ* = 0.46–0.73). Food frequency classification agreement reached 91.6%, depending on the format and population group. These results support the robustness and cultural adequacy of the KomPAN tool for studies conducted in Polish populations (56). Another advantage of the study is related to the inclusion of a wide range of sociodemographic factors and the analysis of the interactions between variables, which allowed for a more comprehensive understanding of the mechanisms affecting the quality of the diet. Conducting latent class analysis was particularly valuable, as it facilitated the identification of various nutritional profiles and groups at an increased risk of adverse dietary habits.

Despite its significant advantages, the study is not devoid of limitations. Its cross-sectional nature does not allow the determination of the direction of cause and effect relationships, which means that it is impossible to clearly determine whether it is nutritional knowledge that affects the quality of the diet, or whether better eating habits are conducive to expanding knowledge in this area. Moreover, the study did not comprise other important factors, such as the level of physical activity, the availability of food in the place of residence or psychological aspects that might additionally affect the quality of the diet. Ultimately, although the multivariate analysis revealed significant relationships between the selected variables, it is possible that some effects were moderated by other environmental or cultural factors not included in the study. Additionally, although self-reported dietary intake is widely used in population-based studies, it is susceptible to recall bias and the effects of social desirability, particularly with regard to sensitive health behaviors such as alcohol consumption and smoking. It is important to note that self-assessed financial status may also be susceptible to subjective interpretation and reporting bias.

Additionally, both financial status and dietary intake were self-reported, which introduces a risk of recall bias and social desirability effects, particularly in relation to sensitive health behaviors such as alcohol consumption and smoking. Ultimately, although the multivariate analysis revealed significant relationships between the selected variables, it is possible that some effects were moderated by other environmental or cultural factors not included in the study.

## 5 Conclusion

The present findings highlight the complexity of factors influencing dietary behaviors and underscore that nutritional education alone is insufficient for achieving substantial improvements in diet quality. Therefore, public health interventions have to integrate strategies addressing the broader socioeconomic context, age-related dietary preferences, and behavioral patterns. Policymakers and public health professionals are encouraged to develop targeted and tailored interventions, with particular emphasis on younger men and populations with lower educational attainment. Effective programs should not only enhance nutritional literacy but also actively support the development of health-promoting behaviors and facilitate informed dietary choices. Moreover, interventions aimed at older adults should acknowledge the stability and cultural embeddedness of their dietary habits, suggesting a need for context-sensitive approaches that respect established food preferences. Implementing these multifaceted strategies has the potential to significantly reduce dietary inequalities and improve overall nutritional health across diverse population subgroups.

## Data Availability

The original contributions presented in the study are included in the article/Supplementary material, further inquiries can be directed to the corresponding author.
