# Supplementary material for: Nutritional knowledge, sociodemographic, and lifestyle factors as determinants of diet quality – a Polish population-based study
Source: Front Public Health. 2025 Aug 21;13:1613598. doi: 10.3389/fpubh.2025.1613598 (PMC12408304; doi:10.3389/fpubh.2025.1613598)
Supplement: Supplementary file 1 [file Table_1.docx]

Supplementary Material 1

# Detailed data on responses to individual nutrition knowledge

| **Statement** | **N** | **%** |
| --- | --- | --- |
| 1. It is enough to eat wholegrains/cereals once a day |  |  |
| True | 2056 | 51.4 |
| False | 1501 | 37.5 |
| I don't know | 443 | 11.1 |
| 1. Only children and adolescents should drink milk |  |  |
| True | 1048 | 26.2 |
| False | 2632 | 65.8 |
| I don't know | 320 | 8 |
| 1. Fruit and/or vegetables should be consumed with every meal |  |  |
| True | 2994 | 74.9 |
| False | 723 | 18.1 |
| I don't know | 283 | 7.1 |
| 1. High intakes of salt protect from hypertension |  |  |
| True | 768 | 19.2 |
| False | 2870 | 71.8 |
| I don't know | 362 | 9 |
| 1. Limiting high-fat foods in everyday diet is protective against cardiovascular diseases |  |  |
| True | 2962 | 74.1 |
| False | 762 | 19.1 |
| I don't know | 276 | 6.9 |
| 1. Frequent consumption of oily fish contributes to atherosclerosis |  |  |
| True | 1052 | 26.3 |
| False | 2244 | 56.1 |
| I don't know | 704 | 17.6 |
| 1. Frequent consumption of grilled meats contributes to the onset of cancer |  |  |
| True | 2511 | 62.8 |
| False | 981 | 24.5 |
| I don't know | 508 | 12.7 |
| 1. Bio-yoghurts contain beneficial gut bacteria |  |  |
| True | 3210 | 80.3 |
| False | 498 | 12.4 |
| I don't know | 292 | 7.3 |
| 1. Vegetable oils and olive oil contain a high amount of cholesterol |  |  |
| True | 1484 | 37.1 |
| False | 1993 | 49.8 |
| I don't know | 523 | 13.1 |
| 1. Wholemeal bread have more fibre than white bread |  |  |
| True | 3147 | 78.7 |
| False | 443 | 11.1 |
| I don't know | 410 | 10.3 |
| 1. Fruit and vegetables are a source of ‘empty calories’ |  |  |
| True | 807 | 20.2 |
| False | 2856 | 71.4 |
| I don't know | 337 | 8.4 |
| 1. Butter and fortified margarines have high content of vitamin A and D |  |  |
| True | 3034 | 75.8 |
| False | 535 | 13.4 |
| I don't know | 431 | 10.8 |
| 1. Cheese is a better source of calcium than cottage cheese |  |  |
| True | 1579 | 39.5 |
| False | 1765 | 44.1 |
| I don't know | 656 | 16.4 |
| 1. Offal has high amounts of ‘bad’ cholesterol - LDL |  |  |
| True | 2267 | 56.7 |
| False | 1047 | 26.2 |
| I don't know | 686 | 17.2 |
| 1. In a healthy diet, complex carbohydrates should be replaced with simple sugars |  |  |
| True | 1756 | 43.9 |
| False | 1299 | 32.5 |
| I don't know | 945 | 23.6 |
| 1. In a balanced diet, proteins should be the main source of energy |  |  |
| True | 2897 | 72.4 |
| False | 665 | 16.6 |
| I don't know | 438 | 10.9 |
| 1. Sun exposure increases the synthesis of vitamin D in the human body |  |  |
| True | 3413 | 85.3 |
| False | 335 | 8.4 |
| I don't know | 252 | 6.3 |
| 1. Consumption of fruit with high content of vitamin C increases bioavailability of iron |  |  |
| True | 2773 | 69.3 |
| False | 535 | 13.4 |
| I don't know | 692 | 17.3 |
| 1. Starting cooking vegetables in cold water helps to preserve the nutrients. |  |  |
| True | 2033 | 50.8 |
| False | 1287 | 32.2 |
| I don't know | 680 | 17 |
