# Supplementary material for: Nutritional knowledge, sociodemographic, and lifestyle factors as determinants of diet quality – a Polish population-based study
Source: Front Public Health. 2025 Aug 21;13:1613598. doi: 10.3389/fpubh.2025.1613598 (PMC12408304; doi:10.3389/fpubh.2025.1613598)
Supplement: Supplementary file 2 [file Table_2.docx]

Supplementary Material 2

# Latent Class Analysis (LCA) Model Fit Evaluation

Supplementary material provides detailed information on the model fit evaluation process used to determine the optimal number of latent classes. A series of LCA models specifying two to five classes was estimated, and model performance was compared using standard fit indices, including the Akaike Information Criterion (AIC), Bayesian Information Criterion (BIC), Sample-Size Adjusted BIC (SABIC), and entropy.

| Model fit | | | | | | | | | | | | |
| --- | --- | --- | --- | --- | --- | --- | --- | --- | --- | --- | --- | --- |
| **Class** | **Log-likelihood** | **Resid.df** | **AIC** | **AIC3** | **BIC** | **SABIC** | **CAIC** | **Entropy** | **G²** | **G² p** | **χ²** | **χ² p** |
| 2 | -22629 | 16359 | 45307 | 45331 | 45458 | 45381 | 45482 | 0.671 | 2301 | 1 | 3043 | 1 |

| **Class** | **Log-likelihood** | **Resid.df** | **AIC** | **AIC3** | **BIC** | **SABIC** | **CAIC** | **Entropy** | **G²** | **G² p** | **χ²** | **χ² p** |
| --- | --- | --- | --- | --- | --- | --- | --- | --- | --- | --- | --- | --- |
| 3 | -21870 | 16346 | 43814 | 43851 | 44047 | 43929 | 44084 | 0.664 | 872 | 1 | 878 | 1 |

| **Class** | **Log-likelihood** | **Resid.df** | **AIC** | **AIC3** | **BIC** | **SABIC** | **CAIC** | **Entropy** | **G²** | **G² p** | **χ²** | **χ² p** |
| --- | --- | --- | --- | --- | --- | --- | --- | --- | --- | --- | --- | --- |
| 4 | -22079 | 16333 | 44258 | 44308 | 44572 | 44413 | 44622 | 0.818 | 1087 | 1 | 1037 | 1 |

| **Class** | **Log-likelihood** | **Resid.df** | **AIC** | **AIC3** | **BIC** | **SABIC** | **CAIC** | **Entropy** | **G²** | **G² p** | **χ²** | **χ² p** |
| --- | --- | --- | --- | --- | --- | --- | --- | --- | --- | --- | --- | --- |
| 5 | -22349 | 16320 | 44823 | 44886 | 45220 | 45020 | 45283 | 0.871 | 1542 | 1 | 1626 | 1 |

| Model comparison | | | | | | | | |
| --- | --- | --- | --- | --- | --- | --- | --- | --- |
| **Class** | **AIC** | **AIC3** | **BIC** | **SABIC** | **CAIC** | **Log-likelihood** | **χ²** | **G²** |
| 1 | 46219 | 46230 | 46289 | 46254 | 46300 | -23099 | 3835 | 2654 |
| 2 | 44486 | 44510 | 44637 | 44561 | 44661 | -22219 | 1000 | 997 |
| 3 | 43811 | 43848 | 44044 | 43926 | 44081 | -21868 | 881 | 870 |
| 4 | 44247 | 44297 | 44562 | 44403 | 44612 | -22073 | 1033 | 1082 |
| 5 | 44823 | 44886 | 45220 | 45020 | 45283 | -22349 | 1626 | 1542 |

**Key Observations:**

- The AIC, BIC, and SABIC values decrease from the two-class to the three-class model, suggesting improved model fit.
- The three-class model has the lowest AIC (43,814) and BIC (44,047), indicating better fit than the two-class model.
- The four-class model shows an increase in BIC and AIC, suggesting possible overfitting.
- The five-class model does not improve fit substantially, as indicated by an increase in AIC and BIC.
- Entropy, which measures classification certainty, increases with more classes (from 0.664 in the three-class model to 0.871 in the five-class model). However, higher entropy alone does not justify selecting a more complex model if other fit indices do not improve.

**Final Model Selection**

- The three-class model appears to be the optimal solution, as it minimizes AIC and BIC while maintaining a reasonable entropy value (0.664).
- The four- and five-class models do not provide significant improvements in model fit while increasing complexity.

**Conclusion**

The three-class solution was selected as the optimal model. It demonstrated the lowest AIC (43,814) and BIC (44,047) values, indicating superior fit relative to models with fewer or more classes. Although the four- and five-class models exhibited higher entropy values, suggesting increased classification certainty, they did not offer meaningful improvements in model fit and were considered less parsimonious. Therefore, the three-class model was retained as it provided the best balance between statistical adequacy and interpretability.
